# CNV-Hub: an integrated web-based platform for CNV classification and interpretation using multi-algorithm consensus

**DOI:** 10.1093/nargab/lqag001

**Published:** 2026-04-23

**Authors:** Vignesh Guru Victor Pillay, Anne-Laure Mosca, Davide Callegarin, Nathalie Marle, Tristan Moro, Mélodie Opale, Nada Maaziz, Gregory Egea, Muriel Payet, Clémence Ragon, Julia Mosnier, Alison Bouzenard, Ludwig Serge Aho, Laurence Faivre, Patrick Callier

**Affiliations:** Department of Chromosomal and Molecular Genetics, Dijon University Hospital, Dijon, 21000, France; Department of Chromosomal and Molecular Genetics, Dijon University Hospital, Dijon, 21000, France; Department of Chromosomal and Molecular Genetics, Dijon University Hospital, Dijon, 21000, France; Department of Chromosomal and Molecular Genetics, Dijon University Hospital, Dijon, 21000, France; Department of Chromosomal and Molecular Genetics, Dijon University Hospital, Dijon, 21000, France; Department of Chromosomal and Molecular Genetics, Dijon University Hospital, Dijon, 21000, France; Department of Chromosomal and Molecular Genetics, Dijon University Hospital, Dijon, 21000, France; Laboratoire de Génétique, GenBio-Inovie, Clermont-Ferrand, 63000, France; Department of Chromosomal and Molecular Genetics, Dijon University Hospital, Dijon, 21000, France; Department of Chromosomal and Molecular Genetics, Dijon University Hospital, Dijon, 21000, France; Department of Chromosomal and Molecular Genetics, Dijon University Hospital, Dijon, 21000, France; Department of Chromosomal and Molecular Genetics, Dijon University Hospital, Dijon, 21000, France; Department of Epidemiology, Dijon University Hospital, Dijon, 21000, France; Department of Clinical Genetics, Dijon University Hospital, Dijon, 21000, France; Department of Chromosomal and Molecular Genetics, Dijon University Hospital, Dijon, 21000, France

## Abstract

Copy number variations (CNVs) are genomic alterations that can cause rare genetic diseases. Their interpretation requires consulting multiple databases and following classification guidelines, such as those from the American College of Medical Genetics (ACMG) and the Clinical Genome Resource (ClinGen). In France, the Achro-Puce working group has also established recommendations to support CNV interpretation. Despite these resources, CNV analysis remains time-consuming, as it requires reviewing gene content, regulatory elements, and associated syndromes. To address this challenge, we developed CNV-Hub, a web-based platform that streamlines CNV classification and interpretation. CNV-Hub integrates five algorithms: two based on ACMG recommendations (AnnotSV, ClassifyCNV), two using machine learning (X-CNV, ISV), and one specifically developed according to French guidelines. In addition to automated pathogenicity predictions, CNV-Hub provides annotations for each CNV, including gene dosage sensitivity scores (pHaplo, pTriplo), syndrome associations, and direct links to databases such as OMIM and PubMed. The platform’s user-friendly interface enables rapid, evidence-based CNV evaluation. By incorporating machine learning among its classification algorithms, CNV-Hub improves the interpretation of uncertain variants by integrating additional parameters. This tool reduces the time required for CNV analysis while maintaining accuracy and reliability, representing a significant advance in molecular cytogenetics and supporting geneticists in clinical decision-making.

## Introduction

Copy number variations (CNVs) are structural genomic variations involving gains and losses of DNA segments. Most CNVs are benign and each individual carries multiple CNVs across their genome [1]. However, some CNVs may overlap one or more dosage-sensitive genes, leading to genetic disorders due to gene deletion or duplication. In addition to dosage effects, CNVs can also exert pathogenic consequences by disrupting gene structure, such as breaking within exons or introns, or by altering regulatory regions such as promoters or enhancers, thereby impairing gene expression. Depending on the gene involved and its mode of inheritance, CNVs may be associated with autosomal dominant disorders, in which a single altered copy is sufficient to cause disease. In other cases, CNVs may contribute to autosomal recessive conditions, where both alleles must be affected, either through two independent CNVs or a combination of a CNV and another type of pathogenic variant on the second allele.

CNV detection and analysis are routinely recommended in medical genetics [2, 3], particularly for the evaluation of individuals with neurodevelopmental disorders (NDDs), multiple congenital anomalies, or fetal ultrasound abnormalities [2]. CNVs are commonly detected using microarray-based techniques such as array-based comparative genomic hybridization (aCGH) or single-nucleotide polymorphism (SNP) arrays but can also be identified through next-generation sequencing (NGS) approaches, including exome and genome sequencing.

Once detected, CNVs require careful interpretation to determine their potential clinical impact. This manual interpretation process, typically performed by experienced cytogeneticists, involves extensive consultation of genomic databases and a meticulous review of relevant scientific literature, with integration of both genomic evidence and the patient’s clinical context. To ensure standardized interpretation, laboratory geneticists rely on established international and national reference guidelines.

The most widely adopted system is the five-tier classification framework proposed by the American College of Medical Genetics and Genomics (ACMG) [4], which categorizes CNVs as Class 1 (benign), Class 2 (likely benign), Class 3 (Variants of Uncertain Significance, VUS), Class 4 (likely pathogenic), and Class 5 (pathogenic).

In the United States, the ACMG and the Clinical Genome Resource (ClinGen) have published technical standards for the interpretation and reporting of germline CNVs [5]. These recommendations are based on a detailed semi-quantitative scoring matrix, with separate tables and criteria for deletions and duplications. This system evaluates multiple parameters, such as gene content, variant frequency, or phenotype correlation, and assigns a classification within the widely adopted five-tier ACMG framework. While comprehensive, their implementation in routine practice can be challenging due to the large number of parameters that must be evaluated.

**Figure 1.**
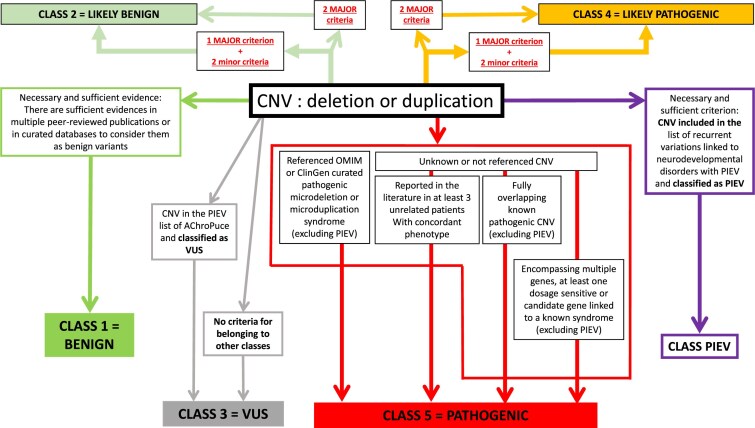
Schematic view of CNVs’ classification according to Achro-Puce Network’s guidelines of 2022. Abbreviations: CNV, Copy Number Variations; PIEV, Incomplete Penetrance and Variable Expressivity; VUS, Variant of Uncertain Significance.

**Figure 2.**
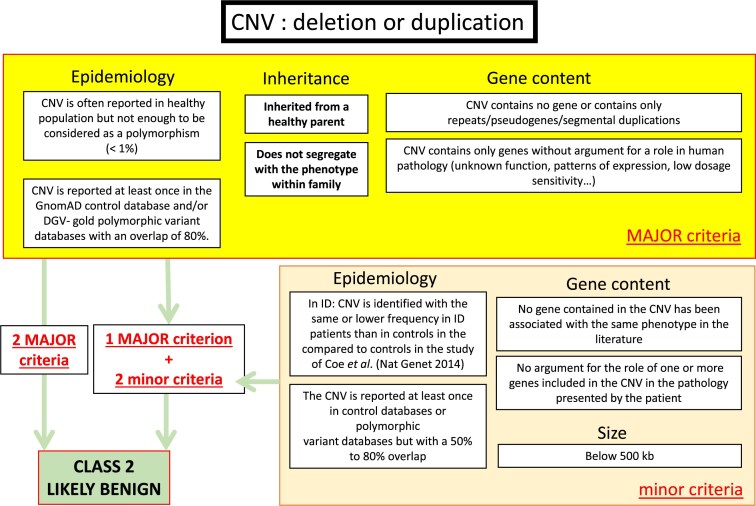
Criteria needed for Class 2 attribution according to Achro-Puce Network’s guidelines of 2022. Abbreviations: GnomAD, Genome Aggregation Database; DGV-gold, Database of Genomic Variants Gold database.

**Figure 3.**
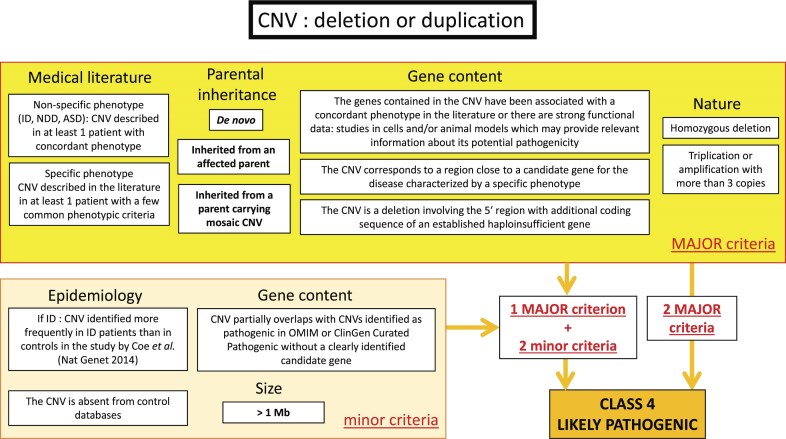
Criteria needed for Class 4 attribution according to Achro-Puce Network’s guidelines of 2022. Abbreviations: ID, Intellectual Deficiency; NDD, Neurodevelopmental Disorders; ASD, Autism Spectrum Disorders.

In parallel, in France, the Achro-Puce network [6], national expert group in molecular cytogenetics, regularly publishes CNV classification guidelines [7, 8]. These provide a unified framework that applies equally to deletions and duplications, and while covering the same five-tier classification as the ACMG system, they adopt a more pragmatic approach with fewer parameters. Their recommendations are summarized in Figs 1–3. A distinctive feature is the addition of a sixth category for recurrent CNVs associated with NDDs showing incomplete penetrance and variable expressivity (PIEV) [8–10]. The PIEV category is integrated into the general interpretation framework and is unique to Achro-Puce. Variants classified in this category are associated with a broad phenotypic spectrum ranging from asymptomatic presentations to severe disease, even within the same family [11]. The official list of recurrent PIEV CNVs, updated annually on the Achro-Puce website, is based on the latest scientific evidence [12].

Although both systems aim to standardize CNV interpretation, their application remains time-consuming due to the need for extensive cross-referencing of databases and literature. This has led to the development of computational tools designed to assist in CNV pathogenicity prediction. We studied four state-of-the-art annotation systems: AnnotSV [13, 14], ClassifyCNV [15], X-CNV [16], and ISV [17]. These tools often yield divergent outcomes due to differences in their decision-making logic and the data they use. As a result, cross-validation between tools is necessary to improve classification reliability. While these algorithms provide valuable first-line support, final interpretation still requires expert manual review to integrate all available genomic and clinical evidence into a conclusive diagnostic report.

To streamline this process, we developed CNV-Hub, a web-based platform that centralizes CNV interpretation resources in a single ergonomic interface (cnvhub.net). CNV-Hub integrates five CNV classification algorithms, curated patient and control databases, detailed gene content with predictive scores, and direct access to key external resources such as genome browsers, the Human Protein Atlas [18], PanelApp [19], SFARI [20], OMIM [21], DECIPHER [22], and Orphanet [23]. This comprehensive platform is designed to improve the speed, accuracy, and consistency of CNV analysis in routine clinical practice.

## Materials and methods

### CNV pathogenicity prediction with computational approaches: state of the art

Several computational tools have been developed to predict the pathogenicity of CNVs, each relying on distinct criteria and algorithmic frameworks. Notable tools include AnnotSV [13, 14], ClassifyCNV [15], X-CNV [16], and ISV [17]. AnnotSV is an integrated annotation tool for structural variants, featuring a web-based interface that applies ACMG–ClinGen classification criteria. ClassifyCNV is another annotation tool also based on ACMG guidelines, offering clinical-grade CNV evaluation. In contrast, X-CNV is a machine-learning-based classifier that was developed with a gradient boosting method [24]. It integrates multiple features, including multiple molecular metrics and CNV-specific attributes (deletion, duplication, length…). ISV is another tool developed with AI and the same gradient boosting approach as X-CNV but emphasizes different features, such as gene content, regulatory elements, and chromatin regions.

As mentioned in the “Introduction” section, cross-checking results from multiple tools is essential for reliable classification. AnnotSV, ClassifyCNV, ISV, and X-CNV are available on GitHub and have been integrated into CNV-Hub to provide reliable and rapid CNV pathogenicity predictions.

We developed a new algorithm based on the French Achro-Puce recommendations that includes the PIEV classification category: “CNV-Hub Achro-Puce algorithm.” This novel algorithm has been implemented on our web-based platform CNV-Hub alongside the four existing tools.

Thus CNV-Hub now provides two algorithms based on ACMG-Clingen guidelines (AnnotSV and ClassifyCNV), two AI-based algorithms (ISV and X-CNV), and one algorithm based on Achro-Puce criteria designed to handle PIEV classification (CNV-Hub Achro-Puce algorithm).

### Development of a new algorithm: “CNV-Hub Achro-Puce,” based on guidelines considering PIEV variants

We developed the first CNV classification algorithm based on the French Achro-Puce network recommendations, with the aim of translating them into a computational and automated framework. To achieve this, we first reconstructed the original Achro-Puce decision trees in Figs 1–3, compiling all criteria and classification logic used by the network. However, not all of these criteria are directly transposable into an algorithm. We therefore performed a selection and simplification process, retaining only the most reproducible, objective, and automatable criteria.

**Figure 4.**
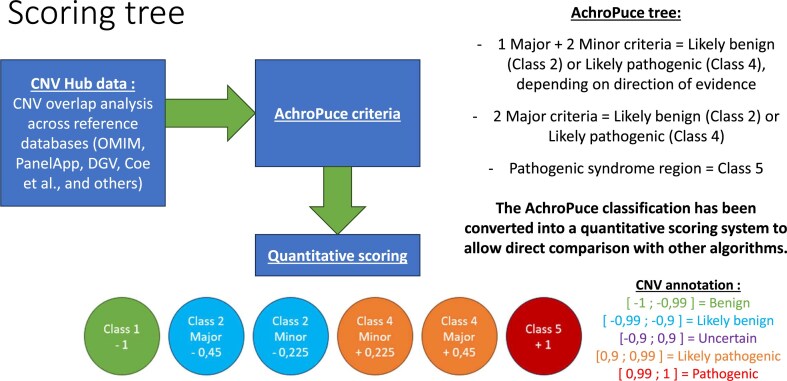
CNV-Hub algorithm scoring system. Abbreviations: OMIM, Online Mendelian Inheritance in Man online catalogue of human genes and genetic disorders; DGV, Database of Genomic Variants.

The list of criteria from the original Achro-Puce guidelines [7] is shown in Table 1, where those implemented in our algorithm are highlighted in green. We then designed a scoring system that translates these selected criteria into a quantitative model. This system, summarized in Fig. 4, assigns a weight to each criterion, enabling the calculation of a global score for each CNV. For example, a CNV overlapping a known syndrome-associated region receives +1 point, whereas a CNV observed multiple times in control databases such as the Database of Genomic Variants (DGV) receives −1 point. Each major criterion contributes +0.45 points to the score, while each minor criterion contributes +0.225 points.

**Table 1.** List of the criteria from the 6-tier classification of Achro-Puce network

6-tier classification according to AChroPuce	
PIEV	cf. Table 2. Official list of NDD variants with PIEV available on AchroPuce website https://acpa-achropuce.com/
Class 5	
	Strong bibliographical arguments (necessary and sufficient criteria) (literature and/or patient databases)
	OMIM or ClinGen Curated Pathogenic microdeletion or microduplication syndrome
	Not referenced in OMIM or ClinGen but reported in the literature in at least three unrelated patients presenting with a concordant specific phenotype
	Not referenced, but a rare or previously undescribed CNV that completely overlaps a CNV referenced in OMIM or ClinGen Curated Pathogenic (excluding PIEV).
	Not referenced but multigenic CNV containing at least 1 haploinsufficient gene identified as a major gene in a microdeletion/microduplication syndrome referenced in OMIM or ClinGen Curated Pathogenic (except PIEV)
Class 4	2 major criteria or 1 major and 2 minor criteria
M*	CNV described in 1 patient presenting a non-specific phenotype (ID, ASD, etc.) in the literature comparable to that of the index case
M	CNV described in 1 patient presenting several specific phenotypic features in the literature comparable to that of the index case
M	Transmission : *de novo*
M	Transmission : inherited from an affected parent
M	Transmission : inherited from a parent who is a mosaic carrier of the CNV
M	gene contained in the CNV associated with the same phenotype in the literature
M	gene contained in the CNV with a strong argument in favor of its involvement in patient’s phenotype (functional studies, expression…)
M	CNV in a region close to a gene to be involved in the disease by specific clinical signs
M	CNV is a deletion that carries the 5′ region and coding sequences of a haploinsufficient gene.
M	Homozygous deletion
M	Amplification (>3 copies)
m**	CNV more frequent in affected patients than in controls in Coe *et al*. case control study (PMID : 25217958)
m	CNV absent from control databases
m	CNVs partially overlapping CNVs referenced as pathogenic in OMIM or ClinGen Curated Pathogenic without a clear candidate gene
m	Size >1 Mb
Class 3	
	CNV not meeting the criteria for other classes
Class 2	2 major criteria or 1 major and 2 minor criteria
M	CNV regularly reported in the general population but not enough to be considered as a polymorphism (<1%)
M	CNV reported at least once in control databases with >80% overlap
M	Transmission: CNV inherited from a healthy parent or does not segregate with the phenotype within a family
M	CNVs containing no genes or only repeated sequences, pseudogenes, segmental duplications
M	CNVs containing only genes with no argument in favour of a role in human pathology (unknown function, expression, scores)
m	For neurodevelopmental disorders: CNV frequency among affected patients not higher than in controls in the Coe *et al*. case control study (PMID : 25217958)
m	CNV reported at least once in control databases with an overlap between 50% and 80%
m	No gene contained in the CNV has been associated with the same phenotype in literature
m	No argument in favour of the involvement of a gene included in the CNV in the phenotype presented by the patient
m	Size below laboratory’s detection threshold
Class 1	
	strong bibliographical arguments in favour of benign character (necessary and sufficient criterion) (literature or control databases)

*M: Major criterion

**m: minor criterion

NDD: Neurodevelopmental disorders

PIEV: variants with incomplete penetrance and variable expressivity

Items considered in CNV-Hub’s algorithm are highlighted in green.

Based on the total score, CNVs are classified as pathogenic if the score is equal to or greater than +1, likely pathogenic if equal to or greater than +0.9, VUS (variant of uncertain significance) if the score lies between −0.9 and +0.9, likely benign if equal to or lower than −0.9, and benign if equal to or lower than −1. The inheritance pattern is also considered in the classification: CNVs inherited from a symptomatic parent or occurring *de novo* are assigned +0.45 points, whereas CNVs inherited from an asymptomatic parent are assigned −0.45 points, in accordance with Achro-Puce guidelines.

Finally, CNVs listed in the Achro-Puce catalog of recurrent CNVs are classified directly according to the network’s recommendations, independently of the computed score. This list, which is updated annually, was retrieved from the Achro-Puce website [12], and each variant was manually annotated with GRCh37 genomic coordinates based on a thorough literature review, as shown in Table 2.

**Table 2.** List of recurrent CNVs associated with neurodevelopmental disorders (NDD) with incomplete penetrance and variable expressivity (PIEV)

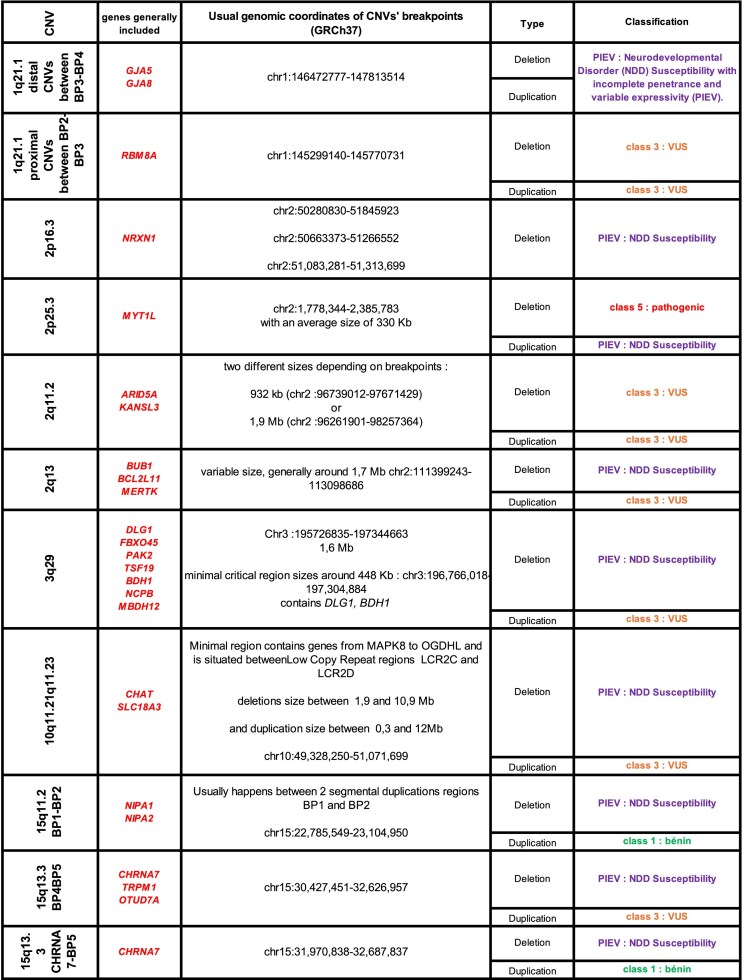 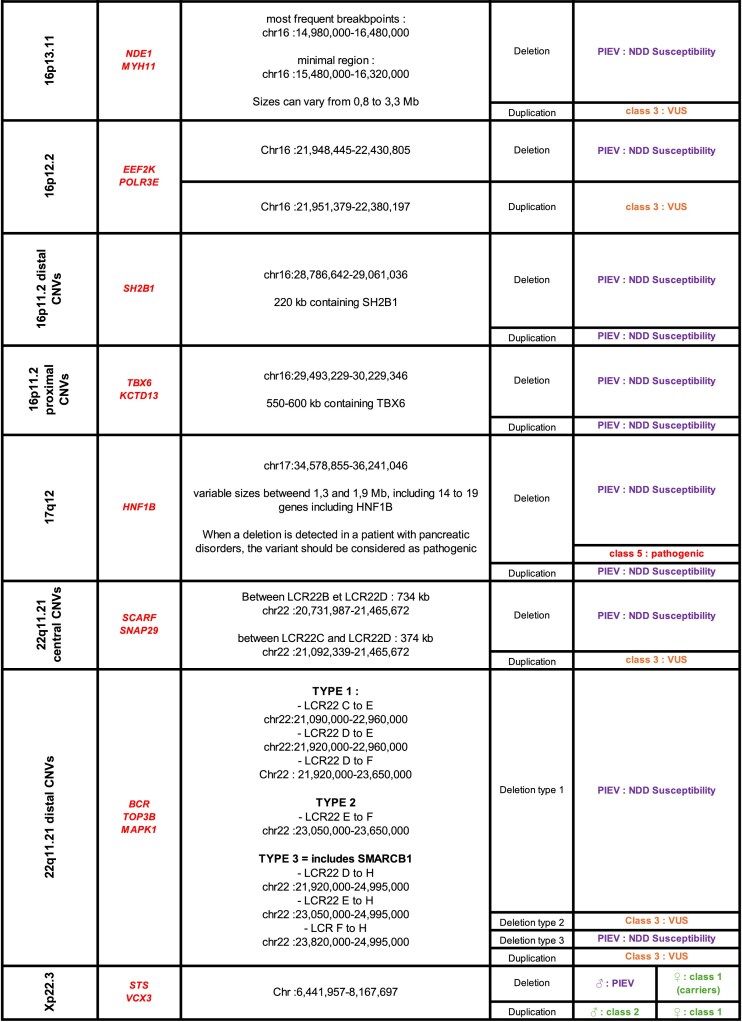

List of variants taken from the 2022 Achro-Puce guidelines and annotated with GRCh37 coordinates for inclusion in the CNV-Hub algorithm.

### Overlap consideration

**Figure 5.**
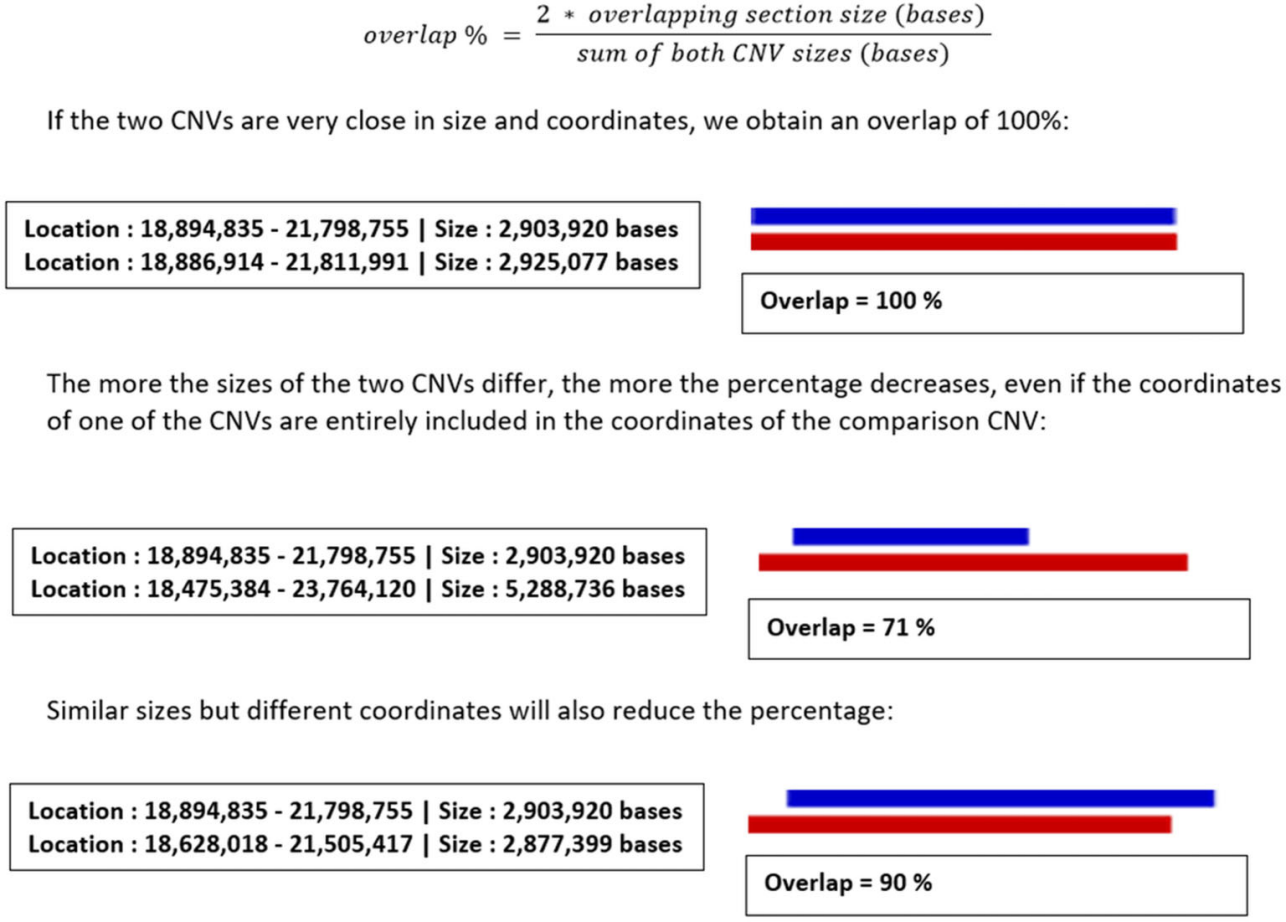
Overlap consideration when comparing patient’s CNV and databases’ CNVs.

CNV-Hub compares the patient’s CNV with entries from reference databases by calculating the percentage of overlap between variants. This overlap is computed as twice the size of the intersecting region divided by the sum of both CNV lengths, as illustrated in Fig. 5. To ensure meaningful comparisons, CNVs showing >30% size difference and with non-overlapping gene content are excluded from the analysis.

The detection of recurrent variants associated with NDDs and listed by the Achro-Puce working group requires consideration of overlap. Indeed, recurrent CNVs occur because of the genomic architecture surrounding them, particularly the presence of segmental duplications or other repetitive elements that predispose rearrangements during meiosis. As a consequence, breakpoints may vary, complicating exact positional matches. To address this, CNV-Hub uses the genomic coordinates of recurrent breakpoints, the positions of associated candidate genes, and the size overlap to accurately classify these variants into the appropriate category as defined by Achro-Puce (Table 2). This approach ensures correct attribution, even when breakpoints slightly deviate from reference positions.

### Implementation and interface development

**Figure 6.**
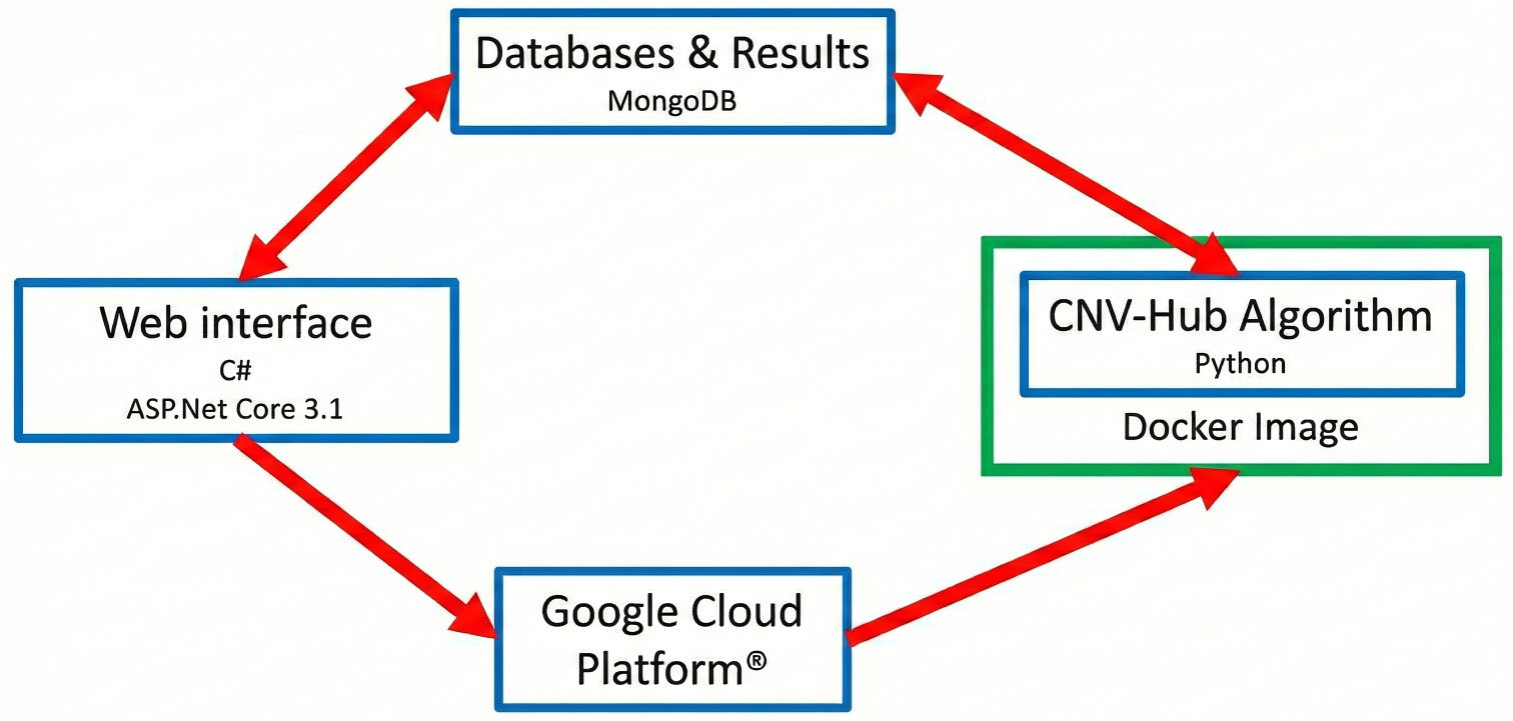
Diagram representing CNV-Hub platform architecture.

The architecture of the CNV-Hub web server is depicted in Fig. 6. The core algorithm, *CNV-Hub Achro-Puce*, which implements the French classification guidelines, was developed in Python 3 and packaged into Docker containers for portability and reproducibility.

The web interface and the pre-loaded reference databases are hosted on a dedicated virtual machine within the Google Cloud Platform (GCP). Computational analyses are executed through containerized Python services, each deployed independently on Google Cloud Run. This architecture cleanly separates the web application from the computation layer, ensuring scalable execution and stable performance.

The platform uses a MongoDB database to manage user queries and to temporarily store intermediate results during each analysis session. No personally identifiable information is collected or retained, and no persistent storage of user-submitted data is implemented in this version.

The user interface was developed in C# using the ASP.NET Core framework, providing a responsive and ergonomic environment for CNV exploration and interpretation. CNV-Hub is a research-focused tool and is not intended for diagnostic use. Its outputs are designed to support variant prioritization but must be interpreted by qualified professionals within a validated clinical framework.

The entire system is hosted on GCP, which provides secure compute isolation and infrastructure aligned with GDPR principles for research-only applications. CNV-Hub is released under a Research-Use-Only (RUO) status and is freely accessible for non-commercial academic use at https://www.cnvhub.net. It is not a certified diagnostic device, and its results must not be used for direct clinical decision-making.

This technological architecture ensures a balance between computational performance, scalability, data protection, and accessibility, enabling rapid and reproducible CNV pathogenicity assessment in a secure research environment.

### Annotation sources: databases

CNV-Hub integrates a wide range of genomic databases that serve as key sources of evidence for CNV interpretation and are directly considered by the classification algorithms. The criteria derived from these resources—such as overlap with known variants—are displayed transparently in the main interface.

For proper CNV interpretation, consulting control population databases is essential. CNV-Hub incorporates the DGV, which is the most comprehensive public resource for structural variants observed in healthy individuals [25]. DGV integrates benign CNV data from multiple studies and databases. The latest DGV data (25 February 2020) were directly downloaded from its official website [26] and allow the algorithm to filter or downgrade variants frequently observed in the general population.

In addition, patient databases provide indispensable information and are considered critical for both American [4] and French classification systems. CNV-Hub integrates several such datasets. The ClinGen database contains CNVs classified as benign, pathogenic, or of uncertain significance and was retrieved via the UCSC Table Browser [27]. CNV-Hub also includes the DECIPHER database [22], which gathers patient CNVs reported by diagnostic laboratories worldwide, along with associated phenotypes and classifications. Furthermore, the platform integrates the morbidity map published by Coe *et al*. in 2014, which compares CNVs from 29 085 children with developmental delay to 19 584 healthy controls [28]. This dataset—also accessible via the UCSC Table Browser—is particularly relevant for applying Achro-Puce criteria: when a CNV is more frequent in cases than in controls, it is considered a minor criterion in favor of pathogenicity; conversely, if more frequent in controls, it supports benignity.

To support gene-level interpretation, CNV-Hub incorporates the latest version of the Reference Sequence (RefSeq) database [29], which provides detailed annotation of transcripts, isoforms, and genomic coordinates. This data, sourced from UCSC Table Browser, enables precise mapping of CNVs onto coding regions. The platform also integrates the HUGO Gene Nomenclature Committee (HGNC) database [30], which ensures that each human gene is unambiguously identified and links known aliases to official gene names, making gene interpretation more robust.

For gene–phenotype correlation, CNV-Hub integrates several specialized resources. The Developmental Disorders Genotype-to-Phenotype (DDG2P) database [31], downloaded from the PanelApp platform, lists genes robustly associated with developmental disorders.

The PanelApp system [32] itself—integrated via an Application Programming Interface (API)—provides community-curated panels with evidence-based gene–disease associations. CNV-Hub includes a dedicated section that lists PanelApp genes falling within each CNV.

Additional phenotype data are retrieved from Online Mendelian Inheritance in Man [21] (OMIM), which catalogs Mendelian disorders and associated genes, and from the Human Phenotype Ontology (HPO) project [33], accessed via API, which links genes to standardized clinical phenotypes.

Lastly, CNV-Hub integrates a curated list of microdeletion and microduplication syndromes to aid syndromic variant detection. The list, published by Wetzel and Darbro in 2022 [34] and available on GitHub, offers a comprehensive literature-based overview of well-characterized syndromic regions, ensuring up-to-date coverage of rare CNV syndromes.

All datasets are reviewed biannually to ensure that the latest versions are utilized.

### Proof-of-concept: CNV selection for testing and model validation

To validate CNV-Hub Achro-Puce algorithm, we had to test a considerable number of CNVs. We initially extracted 3740 benign CNVs from the Gold Database of Genomic Variants (DGV gold) and 39 721 CNVs from ClinGen patient database, with 20 645 benign and 19 076 pathogenic CNVs.

From this dataset of 43 461 CNVs, we randomly selected 2494 pathogenic deletions, 2435 pathogenic duplications, 2500 benign deletions, and 2493 benign duplications using a custom Python script. This random selection allowed us to generate a balanced dataset of 9922 CNVs for testing. This sample was constrained by technical limitations of the platform, which could not process a larger number of variants within a reasonable timeframe. The public interface of CNV-Hub is limited to 300 CNVs per batch. This restriction was bypassed using an internal processing system developed specifically for performance testing.

The new algorithm implementing Achro-Puce criteria was then validated using these curated CNVs. All variants were selected from reliable public databases and had an unambiguous classification. This dataset was used to both validate the performance of CNV-Hub Achro-Puce algorithm and compare it against the four other algorithms integrated within the CNV-Hub platform.

CNV-Hub Achro-Puce algorithm classifies CNVs using the standard five-tier ACMG system and includes a sixth category specific to the French guidelines for classifying recurrent neurodevelopmental susceptibility CNVs (PIEV). However, for this validation step, we simplified the classification system by merging the “likely pathogenic” and “likely benign” categories with “pathogenic” and “benign,” respectively, and none of the tested CNVs belonged to the PIEV category.

CNVs classified as variants of uncertain significance (VUS) by the algorithm were excluded from the analysis. Indeed, when the algorithm lacks sufficient evidence to determine pathogenicity, it assigns the VUS label, which then requires additional expert review by geneticists. Performance was evaluated based on standard statistical measures: sensitivity, specificity, positive predictive value (PPV), and negative predictive value (NPV).

We defined true positives (TP) as pathogenic CNVs correctly classified as pathogenic or likely pathogenic; true negatives (TN) as benign CNVs classified as benign or likely benign; false positives (FP) as benign CNVs misclassified as pathogenic or likely pathogenic; and false negatives (FN) as pathogenic CNVs misclassified as benign or likely benign.

The same statistical validation procedure was applied to CNV-Hub and to each of the four other tools included in the platform, allowing direct comparison of performance.

## Results

### Results for “CNV-Hub Achro-Puce” algorithm

To validate “CNV-Hub Achro-Puce” algorithm based on the French guidelines, we analyzed a total of 9922 CNVs from the DGV Gold and ClinGen databases. Among 4929 pathogenic CNVs, the algorithm correctly identified 3271 true positives (TP), while 8 were classified as false negatives (FN) and 1650 as variants of uncertain significance (VUS). Among 4993 benign CNVs, it correctly classified 1800 as true negatives (TN), 29 as false positives (FP), and 3164 as VUS. After excluding the VUS from the analysis, the algorithm achieved a sensitivity of 99.8%, specificity of 98.4%, positive predictive value (PPV) of 99.1%, and negative predictive value (NPV) of 99.6% (Table 3).

**Table 3.** CNV-Hub model validation; statistical analysis; and comparison with the other state-of-the-art CNV classifying systems

CNV-Hub Achro-Puce algorithm				
CNV	Pathogenic	Benign	PPV	99.1%
positive test : pathogenic class 4–5	3271	29	NPV	99.6%
negative test : benign class 1–2	8	1800		
class 3 : VUS	1650	3164	Sensitivity	99.8%
	4929	4993	Specificity	98.4%
AnnotSV algorithm				
CNV	Pathogenic	Benign	PPV	93.1%
positive test : pathogenic class 4–5	4870	361	NPV	99.6%
negative test : benign class 1–2	3	697		
class 3 : VUS	56	3935	Sensitivity	99.9%
	4929	4993	Specificity	65.9%
ClassifyCNV algorithm				
CNV	Pathogenic	Benign	PPV	99.4%
positive test : pathogenic class 4–5	2283	13	NPV	97.1%
negative test : benign class 1–2	27	906		
class 3 : VUS	2619	4074	Sensitivity	98.8%
	4929	4993	Specificity	98.6%
ISV AI algorithm				
CNV	Pathogenic	Benign	PPV	99.3%
positive test : pathogenic class 4–5	2741	19	NPV	93.4%
negative test : benign class 1–2	313	4406		
class 3 : VUS	1875	568	Sensitivity	89.8%
	4929	4993	Specificity	99.6%
X-CNV AI algorithm				
CNV	Pathogenic	Benign	PPV	90.6%
positive test : pathogenic class 4–5	3733	387	NPV	90.2%
negative test : benign class 1–2	419	3840		
class 3 : VUS	777	766	Sensitivity	89.9%
	4929	4993	Specificity	90.8%

### Comparison with the four other state-of-the-art classification tools

We compared the CNV-Hub Achro-Puce algorithm with four state-of-the-art CNV classification tools: AnnotSV [13, 14], ClassifyCNV [15], and X-CNV [16]. For a fair comparison, we applied the same statistical analysis pipeline used for CNV-Hub to these four tools (Table 3).

Among the tools based on ACMG guidelines: AnnotSV achieved a sensitivity of 99.9% but a low specificity of 65.9%, largely due to a high number of false positives; ClassifyCNV demonstrated more balanced performance, with a sensitivity of 98.8% and specificity of 98.6%. The two AI-based algorithms also performed well: ISV reached a sensitivity of 89.8% and the highest specificity of 99.6%; X-CNV had a sensitivity of 89.9% and a specificity of 90.8%.

While all detection tools demonstrated strong overall performance, CNV-Hub Achro-Puce algorithm distinguished itself by achieving a sensitivity of 99.8%, a specificity of 98.4%, a positive predictive value (PPV) of 99.1%, and a negative predictive value (NPV) of 99.6%. Regarding false classifications: CNV-Hub had 8 false negatives (FN) and 29 false positives (FP); AnnotSV had 3 FN but 361 FP; ClassifyCNV had 27 FN and 13 FP; ISV reported 313 FN and 19 FP; X-CNV had 419 FN and 387 FP. AnnotSV showed the highest sensitivity, explaining its low number of FN, but at the cost of many FP, leading to the lowest specificity. This suggests a tendency to overclassify CNVs as pathogenic.

In terms of variants of uncertain significance (VUS): CNV-Hub classified 33% of pathogenic CNVs (1650/4929) and 63% of benign CNVs (3164/4993) as VUS; AnnotSV: 1% pathogenic (27/4929) and 78% benign (3935/4993); ClassifyCNV: 53% pathogenic (2619/4929) and 81% benign (4074/4993); ISV: 37% pathogenic (1875/4929) and 11% benign (568/4993); X-CNV: 16% pathogenic (777/4929) and 15% benign (766/4993).

These findings confirm the strong performance of “CNV-Hub Achro-Puce” algorithm, not only in terms of classification accuracy, but also in its balance between sensitivity and specificity, and in its capacity to limit overclassification or excessive VUS assignment.

### CNV-Hub user interface

**Figure 7.**
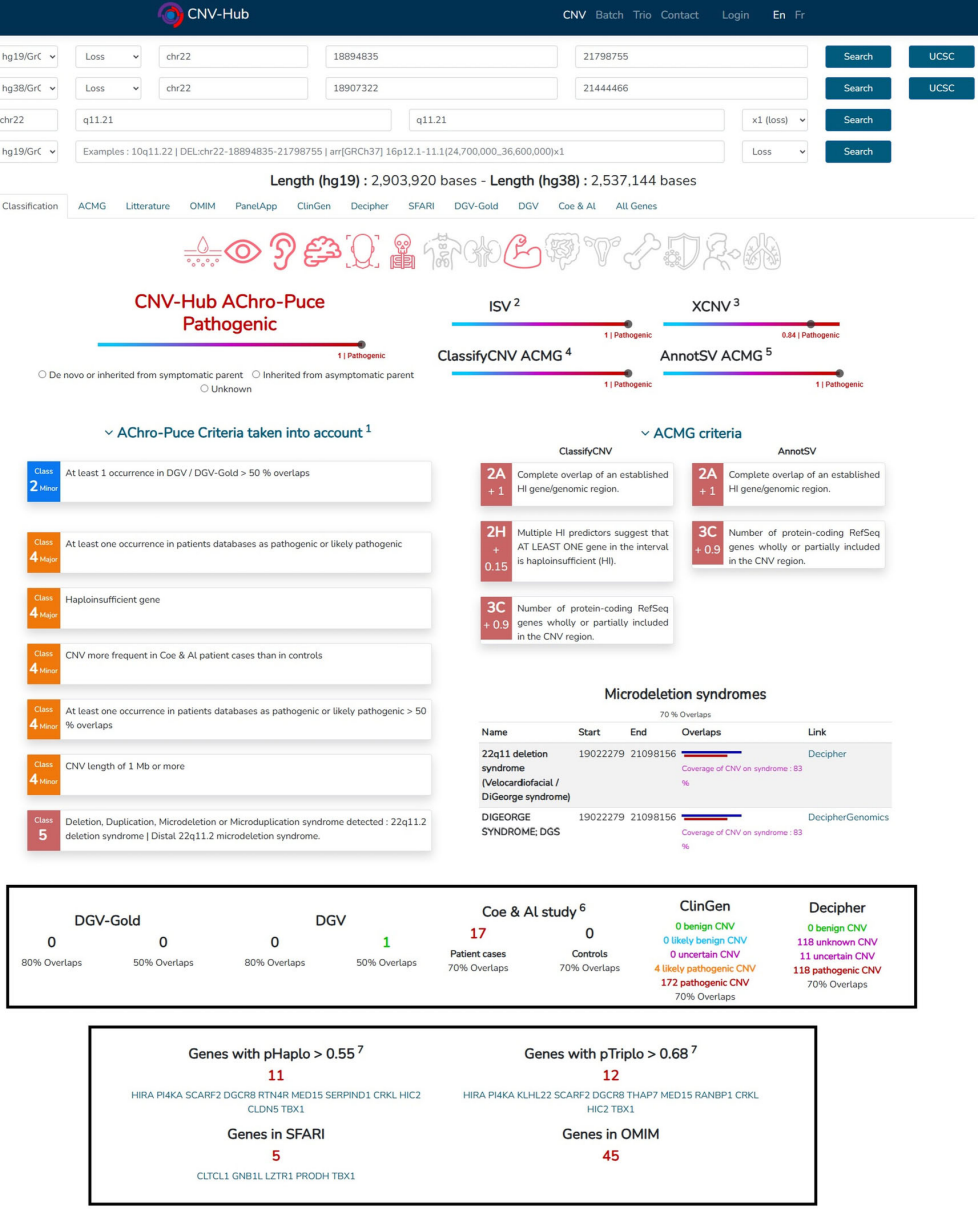
CNV-Hub’s main interface showing an example of a deletion in the 22q11 region associated with DiGeorge syndrome. CNV coordinates are displayed at the top in multiple formats (GRCh37, GRCh38, and cytoband). All five algorithms classify this variant as pathogenic. The expanded view reveals the detailed pathogenicity criteria applied by both the Achro-Puce and ACMG-based algorithms, providing transparency on the factors contributing to the final classification.

The CNV-Hub user interface is designed to be intuitive and versatile, allowing users to input CNV coordinates in various formats, including ISCN nomenclature, chromosomal banding, and UCSC genomic coordinates. The platform automatically translates between genome builds GRCh37 (hg19), GRCh38 (hg38), and chromosomal banding, ensuring compatibility with different types of input data. Once a CNV is submitted, the interface displays its coordinates in all supported formats at the top of the page, followed immediately by the classification results from the five integrated pathogenicity prediction algorithms (Fig. 7). The output from the “CNV-Hub Achro-Puce” algorithm appears first, followed by results from the two machine learning-based tools, X-CNV and ISV, and finally the two ACMG-guideline-based algorithms, ClassifyCNV and AnnotSV. When classifications differ across algorithms, CNV-Hub displays the divergence clearly within the results panel. This allows users to immediately identify variants where guideline-based and machine-learning methods disagree, facilitating rapid prioritization for manual expert reassessment. For the guideline-based tools, CNV-Hub interface presents a detailed list of the criteria used to generate the classification, allowing the user to understand the rationale behind the decision.

A summary section appears at the bottom of the results page, highlighting essential features such as similar variants reported in patient databases, a selection of important genes within the CNV, associated syndromes, and inheritance patterns. This provides users with a first impression of the variant’s clinical relevance before exploring additional details. The interface contains several tabs that enable further exploration of the CNV’s gene content. Four tabs are dedicated to gene categories: OMIM-listed genes, genes from PanelApp panels, genes associated with autism from the SFARI database, and uncharacterized or pseudogenes. Each gene is listed with the number of impacted exons, relevant dosage sensitivity scores (pHaplo, pTriplo), and loss-of-function intolerance scores (pLI, LOEUF). Direct access to external resources is also provided (OMIM, Ensembl, Human Protein Atlas, etc.). For genes associated with disease, the Human Phenotype Ontology (HPO) terms are also provided with their definitions to facilitate phenotype interpretation. Additional tabs provide access to overlapping CNVs reported in public databases, along with their classification and the percentage of overlap. For offline use or downstream analysis, users can export a full list of genes included in the CNV, complete with all annotations, as a downloadable .csv file.

**Figure 8.**
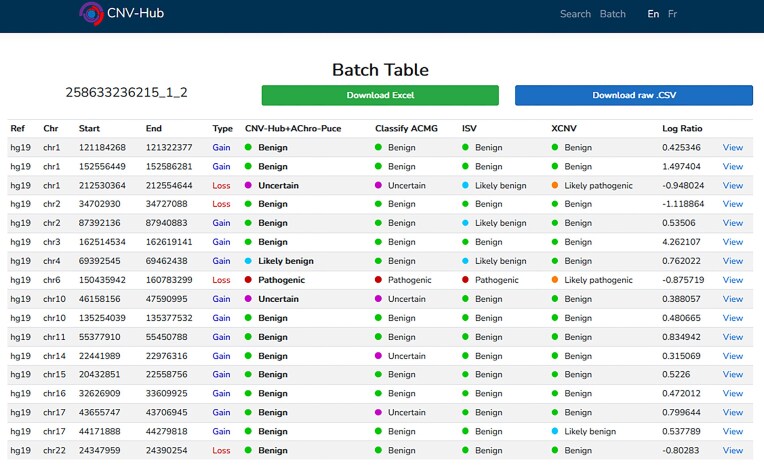
Screen capture from CNV-Hub: example of a batch analysis with the list of CNVs detected on a patient’s sample by microarray. Algorithm predictions appear for each CNV.

CNV-Hub also supports batch analysis, enabling the simultaneous processing of up to 300 CNVs. Variants can be entered manually or uploaded via standardized file formats such as .tsv, .xls, or .txt, facilitating data import directly from detection technologies like microarrays or NGS. This feature can be used either to analyze all CNVs identified in a single patient, or to assess a broader list of CNVs extracted from clinical or research databases. In batch mode, classification results for each variant are displayed alongside clickable links that direct users to the detailed single-CNV analysis interface. This functionality greatly facilitates the identification of clinically significant variants, as illustrated in Fig. 8 with an example based on a complete microarray dataset from one patient. A trio analysis function is also available, allowing users to upload variant lists from a patient and both parents in order to automatically exclude inherited variants. Batch results include classification predictions from all five integrated algorithms, with direct links to detailed single-CNV analysis pages. All results can be exported as spreadsheets for further manual review and filtering.

## Discussion and conclusion

### Multiple objectives of CNV-Hub

CNV-Hub was developed with several objectives in mind. The first was to significantly reduce the time required for CNV pathogenicity interpretation in routine laboratory workflows. This was achieved through an intuitive web-based interface that centralizes key data sources and tools, integrating multiple algorithms that assess diverse genomic features to optimize pathogenicity prediction and allow rapid estimation of clinical impact.

The second objective was to create the first algorithm that would classify recurrent NDD predisposing CNVs with incomplete penetrance and variable expressivity (PIEV). To address this, we developed a new algorithm based on the French Achro-Puce network guidelines, including their recommended decision trees and classification logic. CNV-Hub uses the globally recognized five-tier ACMG system but adds a sixth category, PIEV, as recommended by the French guidelines. CNV-Hub thus combines the globally recognized five-tier ACMG system with a sixth category for recurrent NDD susceptibility CNVs, making it the only platform enabling simultaneous application of French and American standards alongside AI-based prediction tools.

The performance of the CNV-Hub Achro-Puce algorithm is complementary to existing tools. Because each algorithm relies on different criteria and logic trees, their outputs may diverge. However, using multiple classification systems in parallel often reveals consensus patterns or flags discordant results for further review. For instance, AnnotSV, which has the highest sensitivity, tends to overclassify CNVs as pathogenic, while AI-based tools often assign fewer VUS but may show reduced sensitivity and tend to decide more categorically whether they are pathogenic or benign, although they still attribute nearly 15%–25% of uncertain classifications. This variability reinforces the importance of using several tools in combination, a strategy supported by prior studies such as Sládeček *et al*. in 2023 [35], who combined ISV and an algorithm based on ACMG-ClinGen criteria named MarCNV. This reinforces our argument that multiple tools need to be consulted to establish a reliable classification. Importantly, automated tools alone are not sufficient to establish clinical diagnoses. In practice, using several classification systems in parallel is particularly valuable because guideline-based and machine-learning approaches do not rely on the same evidential framework. As a result, each algorithm may classify certain CNVs differently. This variability is expected and reflects the distinct logic trees, feature sets, and training data underlying each method. CNV-Hub was deliberately designed to exploit this complementarity: by presenting all five classifications side-by-side, it enables immediate detection of concordant conclusions as well as rapid identification of discordant variants that require expert review. This multi-algorithm structure is therefore not a limitation, but a functional strength that supports more reliable CNV prioritization and improves the robustness of routine interpretation workflows. In evidence-based medical practice, comprehensive interpretation remains the responsibility of laboratory geneticists, who must also consult patient databases, literature, and gene-specific knowledge to assess CNV relevance.

The third objective of CNV-Hub was thus to provide an integrated interface offering maximum information in one place, enabling efficient and well-informed CNV interpretation. Additional practical features—such as coordinate conversion (hg19, hg38, cytobands)—are tailored for clinical workflows. For example, cytogeneticists working with karyotypes can input banding nomenclature (e.g. 22q11.2) and obtain approximate genomic coordinates to explore gene–phenotype relationships prior to molecular testing.

A fourth goal was to support the reanalysis of variants of uncertain significance (VUS), which represents a major challenge in medical genetics. Many patients remain undiagnosed for years due to inconclusive findings. Although periodic re-evaluation of VUS is recommended [36], it is often impractical due to time constraints. CNV-Hub’s batch analysis feature allows simultaneous evaluation of up to 300 CNVs using a BED-formatted list, making systematic reanalysis feasible on an annual or biennial basis. When confronted with a true VUS, classification results often vary significantly between tools: some algorithms lean toward pathogenicity due to high sensitivity, while others remain uncertain. This variability highlights the necessity of manual review and reinforces the importance of a platform like CNV-Hub that aggregates evidence from multiple tools in one view. By prioritizing speed, completeness, and interpretability, CNV-Hub enables efficient reanalysis, better targeting of suspicious CNVs, and more robust clinical decision-making. This platform supports a practical and scalable approach to ongoing variant interpretation in clinical genomics.

### Limitations of CNV-Hub

While CNV-Hub represents a significant advancement in molecular cytogenetic analysis, several limitations should be acknowledged.

First, when testing well-characterized benign and pathogenic variants in batch mode, a high proportion of variants are classified as variants of uncertain significance (VUS). This was particularly observed with guideline-based algorithms, including CNV-Hub. The main reason is that several criteria from official guidelines cannot yet be fully automated due to current technological constraints. These missing elements require manual review by laboratory geneticists during a second interpretation step. In that sense, the algorithm may be considered to have “failed” when it cannot classify a clearly documented variant without additional human input. In contrast, AI-based algorithms (such as X-CNV and ISV) tend to yield fewer uncertain results. This is likely because they do not rely on clinical guidelines but instead use internal decision frameworks based on molecular features and trained scoring systems. These models provide more categorical outputs and classify most CNVs as either benign or pathogenic. The remaining uncertain classifications correspond to variants for which the model lacks sufficient confidence to assign a definitive label. Their architecture allows full interpretation based on the input data, without requiring additional layers of guideline-based reasoning.

Another limitation is the incomplete coverage of guideline criteria. While users can already input the inheritance mode of a CNV—such as “*de novo*,” “inherited from a symptomatic parent,” or “inherited from an asymptomatic parent”—some other criteria remain unavailable (Table 1). Expanding the interface to allow additional parameters could improve compliance with recommendations, but excessive complexity might deter routine use. Certain rules remain difficult to automate, particularly those requiring a critical review of the literature. Advances in natural language processing and AI may eventually enable automated literature assessment, narrowing the gap between automated and manual interpretation.

Finally, one of CNV-Hub’s key strengths—its integration of multiple algorithms—also reflects a strategic choice. The combination of guideline-based tools (CNV-Hub, ClassifyCNV, AnnotSV) with AI-based algorithms (X-CNV, ISV) enhances performance, as AI tools compensate for some limitations of rule-based systems and provide complementary insights.

### Development prospects

CNV-Hub continues to evolve, with several upcoming features designed to enhance its functionality and usability. In addition, a broader validation phase is planned, ideally through multicenter studies using large and heterogeneous CNV datasets obtained from multiple detection technologies and covering diverse clinical indications. Such studies would compare CNV-Hub classifications with expert consensus and assess key performance metrics such as sensitivity, specificity, positive predictive value, and reproducibility. This will provide robust evidence for its reliability and further guide algorithm refinement for wider clinical use.

One major challenge in CNV analysis is the variability of overlap between variants, which often results in significantly different gene content. To address this, we are developing a gene content comparison tool that will highlight shared and unique genes between overlapping CNVs, helping users to better understand genotype–phenotype correlations and to identify potential candidate genes. In addition, CNV-Hub will soon support the integration of local variant databases, allowing laboratories to query and visualize their own clinical data directly within the platform with anonymized data. A new variant and gene prioritization system is also in development. This feature will allow users to rank variants based on customizable criteria such as gene scores, HPO terms, SFARI scores, and other functional annotations.

These tools will enable users to more easily identify relevant genes and match variants with similar profiles across public and internal datasets. To further improve usability, a redesigned visualization interface is under development, offering enhanced navigation and an even more ergonomic layout. This will facilitate faster interpretation and clearer understanding of CNV pathogenicity. CNV-Hub remains in active development, with the goal of supporting laboratory geneticists in CNV interpretation across various detection methods, including both microarrays and NGS. In particular, its batch analysis mode, which enables the simultaneous analysis of hundreds of CNVs, makes it an ideal tool for periodic reanalysis of VUS.

In conclusion, CNV-Hub represents a major step forward in CNV interpretation, offering a robust, scalable, and integrative platform. By combining multiple classification algorithms and curated data sources, it provides laboratory geneticists with an efficient and comprehensive solution to improve diagnostic accuracy and patient care.

## Data Availability

CNV-Hub is released under a RUO status and is freely accessible for non-commercial academic use at https://www.cnvhub.net.
